# THE IMPORTANCE OF LOW SOCIAL LONELINESS FOR MAINTAINING GOOD WELL-BEING

**DOI:** 10.1093/geroni/igz038.2143

**Published:** 2019-11-08

**Authors:** Lise Switsers, Liesbeth De Donder, Eva Dierckx, Sarah Dury

**Affiliations:** 1 Vrije Universiteit Brussel, Brussels, Belgium; 2 Vrije Universteit Brussel, Brussel, Brussels Hoofdstedelijk Gewest, Belgium

## Abstract

Older people are often confronted with dependence, death of spouse and other loss experience. Nevertheless, older adults generally experience a good well-being. This lack of age-related decline of subjective well-being has been named the ‘paradox of ageing’. One possible explanation for this paradox can be found in the socio-emotional selectivity theory of Carstensen. Thus, we hypothesize that low emotional and/or low social loneliness can act as a buffer for the negative relationship between negative life events and well-being. We use data of the D-SCOPE project that includes 869 older community-dwelling adults at risk of frailty residing in Flanders. By means of regression moderating analyses the research gains insights into the relationships between older people and well-being where the absence of social loneliness is detected as a possible buffer against negative outcomes. The discussion develops the argument that the absence of loneliness is a crucial facet for maintaining a good well-being.

